# The complete chloroplast genome of a Chinese medicinal plant, *Peristrophe japonica* (Thunb.) Bremek. (Lamiales: Acanthaceae) from Nanjing, China

**DOI:** 10.1080/23802359.2021.1934157

**Published:** 2021-06-07

**Authors:** Jingxin Chen, Long Wang, Yucheng Zhao, Minjian Qin

**Affiliations:** School of Traditional Chinese Medicine, China Pharmaceutical University, Nanjing, China

**Keywords:** Chloroplast genome, *Peristrophe japonica*, phylogenetic analysis

## Abstract

*Peristrophe japonica* (Thunb.) Bremek. is a widely distributed medicinal plant species in China and Japan. In this study, the complete chloroplast genome sequence of *P. japonica* was assembled and characterized from high-throughput sequencing data. The chloroplast genome is 151,374 bp in length, consisting of a large single-copy (LSC) and a small single-copy (SSC) regions of 83,395 bp and 17,073 bp, respectively, which were separated by a pair of 25,453 bp inverted repeat (IR) regions. The overall GC content of the genome is 38.07%. The genome contains 133 genes, including 88 protein-coding, 37 tRNA, and eight rRNA genes. A phylogenetic tree reconstructed using 23 chloroplast genomes reveals that *Peristrophe* form a separate group which is a sister of the genus *Dicliptera*. The work reported here is the first complete chloroplast genome of *P. japonica* which will provide useful information to the evolutionary studies on the genus of *Peristrophe*.

*Peristrophe japonica* (Thunb.) Bremek., with little pink flowers and beautiful slender shape, is a ground covering perennial (Chen et al. 2016). *P. japonica* is widely distributed in China and Japan. It is well known in the national minority of China as herbal medicine in daily life, and is also to treat colds and fever (He et al. 2013). Pharmacological analysis shows that *P. japonica* has anti-bacterial, anti-inflammatory, and cough relief efficacy (Li et al. 2010). In addition to medicine, it is successfully used in landscape areas for soil and water conservation. Previous studies on *P. japonica* mainly focused on the medical effect or the cultivation techniques. However, the chloroplast genome information of *P. japonic*a has not been characterized. In this study, the complete chloroplast genome of *P. japonica* was determined using high throughput sequencing technology to contribute to the bioinformatics and evolutionary for the phylogenetics of the genus *Peristrophe*.

The fresh leaves of *P. japonica* were sampled from Nanjing city, Jiangsu Province, China (31°54′10.368″ N, 118°55′5.12″ E). Specimens were stored in the Medicinal Botanical Garden of China Pharmaceutical University (accession number: CPU-JTSZC, Minjian Qin, minjianqin@163.com). Total genomic DNA was extracted with a modified CTAB protocol according to Doyle and Doyle (1987). The whole genome sequencing was conducted by Hefei Biodata Biotechnologies Inc. (Hefei, China) on the Illumina Hiseq platform. The filtered sequences were assembled using the program SPAdes assembler 3.10.0 using the default settings (Bankevich et al. 2012). The annotation was performed with DOGMA (Wyman et al. 2004) and BLAST searches.

The chloroplast genome of *P. japonica* is 151,374 bp in length (GenBank accession no. MW411448), and contains two inverted repeat (IR) regions of 25,453 bp, separated by large single-copy (LSC) and small single-copy (SSC) regions of 83,395 bp and 17,073 bp, respectively. The overall GC content of the *P. japonica* complete chloroplast genome is 38.07% and the corresponding values in LSC, SSC, and IR regions are 36.1%, 32.1%, and 43.3%, respectively. The complete chloroplast genome was predicted to contain 133 genes, including 88 protein-coding, 37 tRNA, and eight rRNA genes. Nine protein-coding, six tRNA, and four rRNA genes were duplicated in the IR regions. Nineteen genes contained two exons and four genes (*clp*P, *ycf*3, and two *rps*12) contained three exons.

To investigate its taxonomic status, alignment was performed on the 23 complete chloroplast genome sequences which are all from Acanthaceae using MAFFT v7.307 (Katoh and Standley 2013), and a maximum-likelihood (ML) tree was constructed with 1000 bootstrap replicates in FastTree v2.1.10 (Price et al. 2010) with the GTR + Gamma model. *Strobilanthes tonkinensis* and *S. cusi*a are as outgroups to construct the phylogenetic tree. As previously published by Deng et al. (2016) *Peristrophe* form a separate group which is a sister of the genus *Dicliptera* (Figure 1). There has no complete chloroplast genome sequence of other species in *Peristrophe* for reference. Hence, more genome studies of other species in *Peristrophe* would be necessary for detailed taxonomy research. The complete chloroplast genome sequence of *P. japonica* will provide a useful resource for the conservation genetics of this species as well as for the phylogenetic studies of the Acanthaceae.

**Figure 1.**
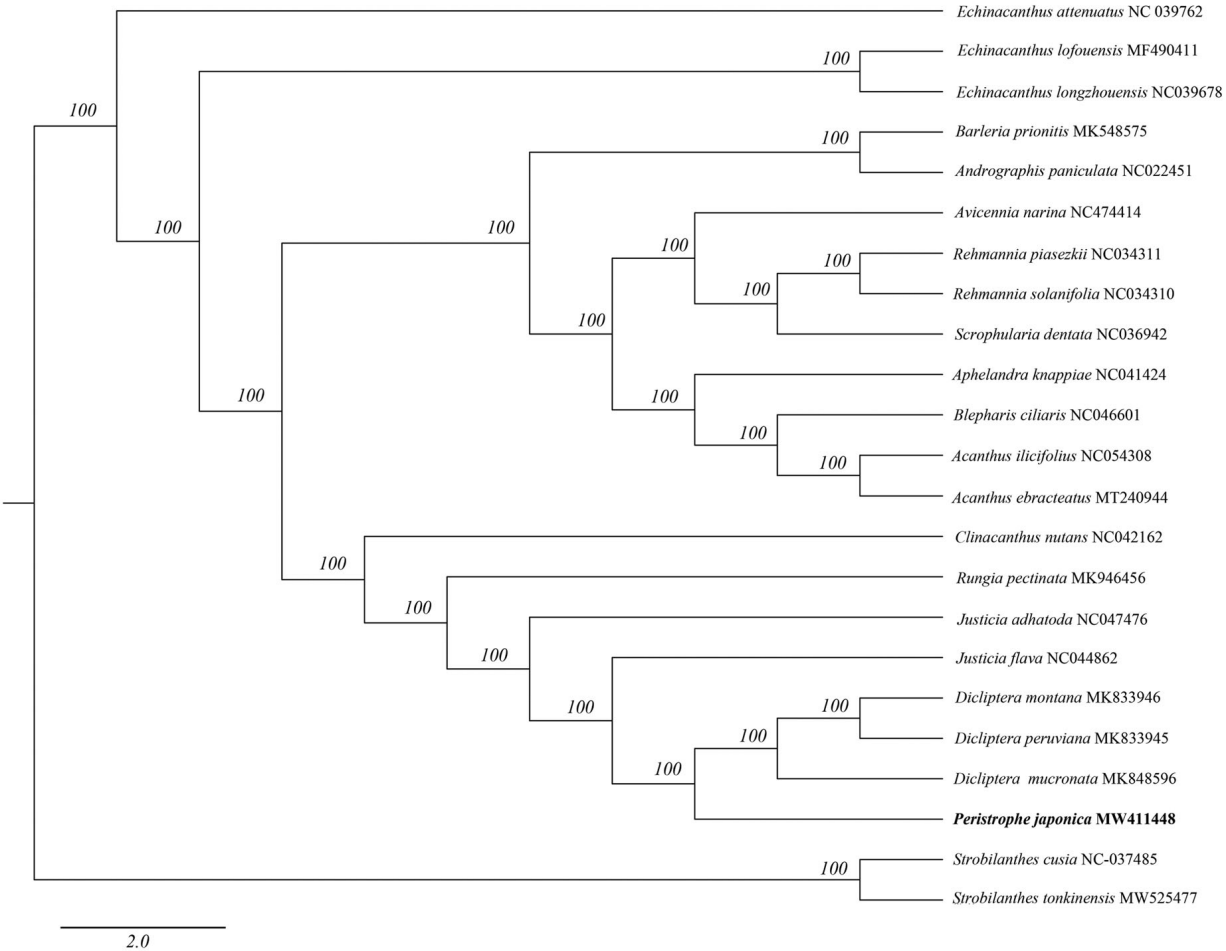
Phylogenetic tree inferred by maximum-likelihood (ML) method based on 23 representative species. A total of 1000 bootstrap replicates were computed and the bootstrap support values are shown at the branches. GenBank accession numbers are displayed along with the plant binomials.

## Data Availability

The genome sequence data that support the findings of this study are openly available in GenBank of NCBI at https://www.ncbi.nlm.nih.gov/ under the accession no. MW411448. The associated BioProject, SRA, and Bio-sample numbers are PRJNA685332, SRS7884003, and SAMN17082919, respectively.
